# Targeted bisulfite sequencing identified a panel of DNA methylation-based biomarkers for esophageal squamous cell carcinoma (ESCC)

**DOI:** 10.1186/s13148-017-0430-7

**Published:** 2017-12-15

**Authors:** Weilin Pu, Chenji Wang, Sidi Chen, Dunmei Zhao, Yinghui Zhou, Yanyun Ma, Ying Wang, Caihua Li, Zebin Huang, Li Jin, Shicheng Guo, Jiucun Wang, Minghua Wang

**Affiliations:** 1 Department of Biochemistry and Molecular Biology, Medical College, Soochow University, Suzhou, Jiangsu China; 2 State Key Laboratory of Genetic Engineering, Collaborative Innovation Center for Genetics and Development, School of Life Sciences and Institutes of Biomedical Sciences, Fudan University, Shanghai, China; 3 Ministry of Education Key Laboratory of Contemporary Anthropology, School of Life Sciences, Fudan University, Shanghai, China; 4 Genesky Biotechnologies Inc., Shanghai, China; 5 Center for Human Genetics, Marshfield Clinic Research Foundation, 9500 Gilman Drive, MC0412, Marshfield, Wisconsin 54449 United States

**Keywords:** Esophageal squamous cell carcinoma, DNA methylation, Biomarker, Diagnosis, Targeted bisulfite sequencing

## Abstract

**Background:**

DNA methylation has been implicated as a promising biomarker for precise cancer diagnosis. However, limited DNA methylation-based biomarkers have been described in esophageal squamous cell carcinoma (ESCC).

**Methods:**

A high-throughput DNA methylation dataset (100 samples) of ESCC from The Cancer Genome Atlas (TCGA) project was analyzed and validated along with another independent dataset (12 samples) from the Gene Expression Omnibus (GEO) database. The methylation status of peripheral blood mononuclear cells and peripheral blood leukocytes from healthy controls was also utilized for biomarker selection. The candidate CpG sites as well as their adjacent regions were further validated in 94 pairs of ESCC tumor and adjacent normal tissues from the Chinese Han population using the targeted bisulfite sequencing method. Logistic regression and several machine learning methods were applied for evaluation of the diagnostic ability of our panel.

**Results:**

In the discovery stage, five hyper-methylated CpG sites were selected as candidate biomarkers for further analysis as shown below: cg15830431, *P* = 2.20 × 10^−4^; cg19396867, *P* = 3.60 × 10^−4^; cg20655070, *P* = 3.60 × 10^−4^; cg26671652, *P* = 5.77 × 10^−4^; and cg27062795, *P* = 3.60 × 10^−4^. In the validation stage, the methylation status of both the five CpG sites and their adjacent genomic regions were tested. The diagnostic model based on the combination of these five genomic regions yielded a robust performance (sensitivity = 0.75, specificity = 0.88, AUC = 0.85). Eight statistical models along with five-fold cross-validation were further applied, in which the SVM model reached the best accuracy in both training and test dataset (accuracy = 0.82 and 0.80, respectively). In addition, subgroup analyses revealed a significant difference in diagnostic performance between the alcohol use and non-alcohol use subgroups.

**Conclusions:**

Methylation profiles of the five genomic regions covering cg15830431 (*STK3*), cg19396867, cg20655070, cg26671652 (*ZNF418*), and cg27062795 (*ZNF542*) can be used for effective methylation-based testing for ESCC diagnosis.

**Electronic supplementary material:**

The online version of this article (10.1186/s13148-017-0430-7) contains supplementary material, which is available to authorized users.

## Background

Esophageal cancer is one of the most aggressive cancers and one of the leading causes of cancer death worldwide [1–3]. Esophageal cancer can be classified as esophageal adenocarcinoma (EAC) or esophageal squamous cell carcinoma (ESCC) by histology [4, 5]. The incidence of EAC is higher in Western countries, while the ESCC subtype is predominant in Asians, especially in China (88.84%), suggesting that the studies of ESCC in the Chinese population is of great importance [6–10]. Currently, most of ESCCs are diagnosed at advanced stages, and studies have revealed that the 5-year survival rate is much higher in the early stage than in the advanced stages of ESCC, indicating the urgent need for effective early diagnosis methods [11–13].

DNA methylation is a key epigenetic modification in the mammalian genomes with many essential functions, including the repression of gene expression and genomic imprinting [14–17]. Numerous studies have suggested that the altered DNA methylation patterns in tumor tissues may silence the tumor suppressor genes and activate the oncogenes through hyper/hypo methylation [18, 19]. In addition, DNA methylation alterations have been found to occur early in the carcinogenesis and therefore could be applied as a promising biomarker for cancer early detection [20–22]. Till now, numerous DNA methylation-based biomarkers have been identified in several types of cancers, including lung cancer, colorectal cancer, prostate cancer, gastric cancer, etc. [23–26]. What is more, *SHOX2* methylation-based screening biomarker has been commercialized in lung cancer [27]. However, despite of several diagnostic panels for ESCC detection, these studies were limited by the relatively small sample size, inaccurate methylation detection methods, and lack of validation datasets. Biomarkers with these limitations may pose a burden for the further prospective studies with large sample sizes.

Therefore, due to the limitations of the current biomarkers, we want to extract more cost-efficient biomarkers with high sensitivity and specificity for ESCC early diagnosis. In addition, with the fast development of liquid biopsy of cancer diagnosis, the diagnostic biomarkers are urgently needed and applied for the large-scale prospective studies. Here, we integrated the ESCC methylation datasets from the public database for biomarker screening and validated a biomarker panel consisting of five candidate CpG sites in 94 pairs of ESCC and normal tissues from the Chinese Han population. Due to the relatively high specificity in ESCC diagnosis, the biomarker panel might be further applied in the liquid biopsy of ESCC along with the other biomarkers with high sensitivity.

## Results

### Integration of TCGA datasets and GEO datasets for biomarker discovery

**Fig. 1:**
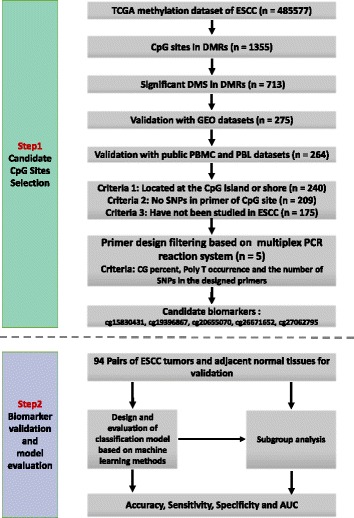
Flow chart of the study design. Candidate biomarkers were selected from the high-throughput DNA methylation microarrays from the TCGA project and further validated with the ESCC methylation data from the GEO dataset, as well as PBMC and PBL from healthy controls. In addition, the PBL and PBMC methylation datasets from healthy samples were also utilized for biomarker filtering. Based on our preliminary screening, the candidate methylation biomarkers for ESCC were then further validated with targeted bisulfite sequencing in independent Chinese Han ESCC patients

Public DNA methylation microarray datasets of ESCC were carefully searched. The esophageal carcinoma methylation dataset from TCGA was first identified, with 84 ESCC tumors and 3 ESCC adjacent normal tissue samples, as well as 78 EAC tumors and 13 EAC adjacent normal tissues. In order to achieve better statistical power, we combined the ESCC and EAC adjacent normal tissues as the control samples due to their similarity, which could be validated using PCA analysis (Additional file 1: Figure S1). As a result, 84 ESCC tumor tissues as well as 16 adjacent normal tissues were employed for the discovery stage analysis. In addition, the GSE52826 dataset from the Gene Expression Omnibus (GEO) database, with a relatively small sample size (4 ESCC tumors and 8 control tissues), was also utilized as the validation dataset [28]. Based on our feature selection procedure and the primer design filtering for constructing the multiplex PCR reaction system, which was described in the “Methods” section (Fig. 1), cg15830431 (*P* = 2.20 × 10^−4^), cg19396867 (*P* = 3.60 × 10^−4^), cg20655070 (*P* = 1.71 × 10^−3^), cg26671652 (*P* = 5.77 × 10^−4^), and cg27062795 (*P* = 3.60 × 10^−4^) were selected for further validation. Among them, cg19396867 and cg20655070 were not in the regulatory regions of specific genes, while cg15830431 (*STK3*, CpG Island), cg26671652 (*ZNF418*, CpG Shore), and cg27062795 (*ZNF542*, CpG Island) were either in CpG islands or the CpG shores of a gene. We showed that these 5 selected CpG sites were significantly hyper-methylated in the ESCC tumor tissues, compared to the adjacent normal tissues. Moreover, the methylation status of these 5 CpG sites was also validated in GSE52826 dataset and showed similar results. In addition, all 5 CpG sites showed hypo-methylated states in the PBMC (peripheral blood mononuclear cells) and PBL (peripheral blood leucocytes) from healthy samples (Additional file 2: Table S1). Based on the above analysis, we believed that these 5 CpG sites would be the candidate non-invasive biomarkers for ESCC. As a result, we built a prediction model based on the logistic regression using all 5 predictors without adjustment for age, gender, and other covariates, which provided a way to discriminate between ESCCs and normal tissues (sensitivity = 0.89, specificity = 0.81, AUC = 0.87). To further evaluate and validate the diagnostic ability of these 5 CpG sites, we then conducted the validation study in 94 paired ESCC and adjacent normal tissue samples obtained from patients from the Chinese Han population.

### Methylation status validation of the five CpG sites with targeted bisulfite sequencing

**Fig. 2:**
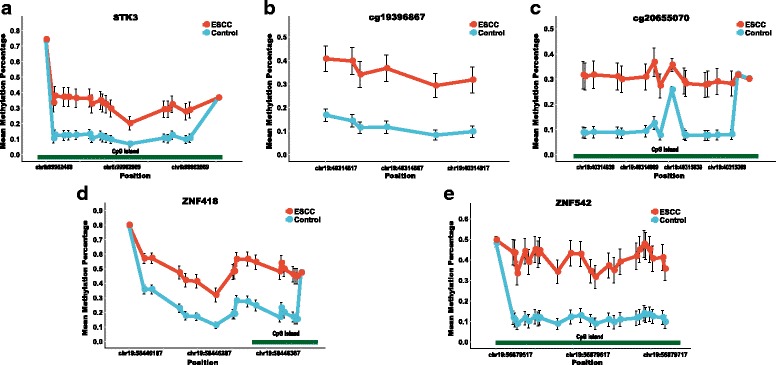
The methylation status of the CpG sites in the five genomic regions. **a**–**e** represent the methylation status of the CpG sites in regions covering *STK3*, cg19396867, cg20655070, *ZNF418*, and *ZNF542*, respectively. The *x*-axis represents the genomic positions of the CpG sites in the targeted regions. The *y*-axis represents the mean methylation percentage in the ESCC tumor tissues as well as the normal tissues for each of the CpG sites. The error bar represents the confidence interval of the methylation percentage in the ESCC tumor tissues as well as the normal tissues for each of the CpG sites

**Fig. 3:**
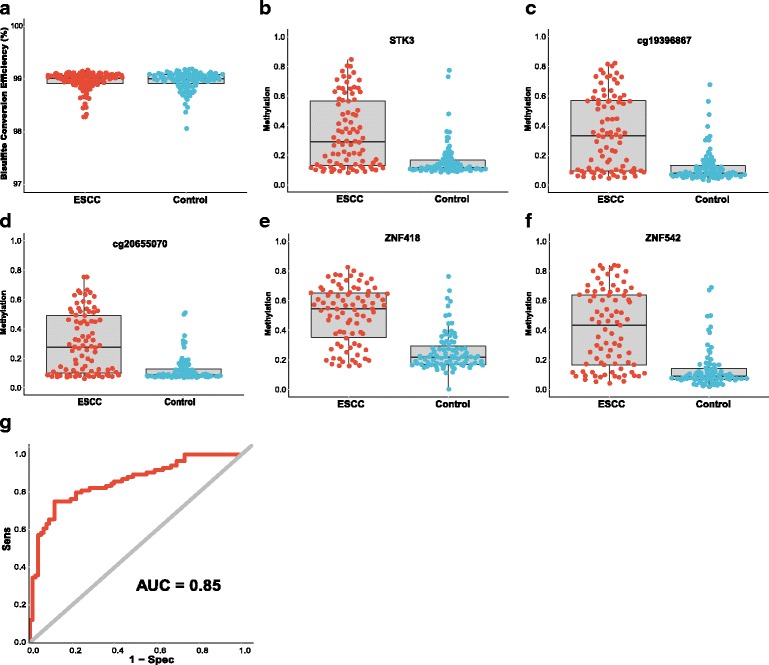
The mean methylation status of each genomic region and bisulfite conversion efficiency between ESCC tumors and normal tissues as well as the overall ROC (Receiver Operating characteristics) curve. **a** represents the bisulfite conversion efficiency between ESCC and adjacent normal tissues. Bisulfite conversion efficiency was calculated by using the number of transformed *C* to *T* divided by the number of *C* in each sample. **b**–**f** represent the mean methylation status of the genomic regions covering *STK3*, cg19396867, cg20655070, *ZNF418*, and *ZNF542*, respectively. Each point represents mean methylation percentage in a genomic region of a sample. The boxplot showed overall methylation percentage of different groups in each genomic region. **g** represents the overall ROC curve, which was calculated through a logistic regression model, incorporating the mean methylation percentage of the five genomic regions as the variables and without the adjustment for gender, age, and smoking status and alcohol status

The characteristics of the ESCC patients are shown in Table 1. Quality control procedures were first applied to the targeted bisulfite sequencing data. We found that the bisulfite conversion rate of each sample was higher than 98%, and no significant difference was found between the tumors and adjacent normal tissues, indicating the bisulfite conversion was efficient and reliable (Fig. 3a). In addition, the samples and the CpG sites with high missing rate (> 30%) and low coverage (< 20×) were also filtered out as described in the “Methods” section. After the quality control procedures, 174 of the 188 samples (94 pairs of ESCC tumor/adjacent normal tissues) still remained for further study. The principal component analysis (PCA) was conducted for all samples and showed a clear discrimination between ESCC tumors and adjacent normal tissues (Additional file 3: Figure S2). Differential methylation analyses were conducted for the five CpG sites as well as nearby CpG sites, suggesting a major difference between the ESCCs and adjacent normal tissues (Fig. 2). A logistic regression model was then applied and showed significant hyper-methylation of the five selected CpG sites in the ESCCs (Table 2, cg15830431, *P* = 1.25 × 10^−6^; cg19396867, *P* = 2.71 × 10^−11^; cg20655070, *P* = 8.04 × 10^−10^; cg26671652, *P* = 4.82 × 10^−11^; cg27062795, *P* = 1.23 × 10^−12^). As a result, we then averaged the methylation status of all the nearby CpG sites in a genomic region as representatives of the candidate regions for further analysis (Fig. 3b–f). Based on the mean methylation status of the five genomic regions, the prediction ability of each region separately was evaluated through logistic regression without adjustment for age, gender, and other covariates. The sensitivity of each region ranges from 0.64 to 0.74, while the specificity ranges from 0.82 to 0.90 and the AUC ranges from 0.76 to 0.84 (Table 3). Moreover, in the logistic model taking all of the five regions as predictors, we obtained the sensitivity of 0.75 and specificity of 0.88, as well as the AUC of 0.85 (Fig. 3g).

**Table 1:** Characteristics of the ESCC patients included in this study

Characteristics	Patient distribution *N* = 94
Age	64 (IQR = 57 to 70)
Sex	
Male	69
Female	25
Cigarette use^a^	
Yes	58
No	36
Alcohol use^b^	
Yes	34
No	58
T stage^c^	
T2	14
T3	72
T4	5
N stage^c^	
N0	44
N1	38
N2	7
N3	3
M stage^c^	
M0	90
M1	1

*ESCC* esophageal squamous cell carcinoma

^a^Yes represents the former and current smokers

^b^Yes represents individuals who presently consume or formerly consumed alcoholic beverages

^c^TNM stages were assessed by the seventh edition of the TNM classification criteria

**Table 2:** The methylation status of the five CpG sites in the TCGA dataset and the validation dataset

	CpG site	Gene	Position	Relation to CpG_Island	McaM^b^	McoM^b^	*P* value^c^	log_10_(OR)^d^	95% CI^d^	Sens	Spec	AUC
TCGA	cg15830431	STK3	chr8:99952591	Island	0.28	0.09	2.20E−04	4.11	1.91–7.43	0.65	0.94	0.82
	cg19396867	NA^a^	chr19:40314862	N_Shore	0.45	0.20	3.60E−04	1.85	0.78–3.21	0.85	0.75	0.79
	cg20655070	NA^a^	chr19:40315011	Island	0.44	0.19	1.71E−03	1.61	0.67–2.72	0.64	0.88	0.75
	cg26671652	ZNF418	chr19:58446312	N_Shore	0.35	0.16	5.77E−04	1.95	0.67–3.61	0.86	0.75	0.78
	cg27062795	ZNF542	chr19:56879613	Island	0.43	0.17	3.60E−04	2.93	1.65–4.44	0.86	0.81	0.80
Validation	cg15830431	STK3	chr8:99952591	Island	0.20	0.07	1.25E−06	3.04	1.82–4.53	0.66	0.77	0.71
	cg19396867	NA	chr19:40314862	N_Shore	0.37	0.12	2.71E−11	2.83	1.93–3.91	0.65	0.88	0.80
	cg20655070	NA	chr19:40315011	Island	0.31	0.09	8.04E−10	3.01	2.02–4.22	0.62	0.89	0.77
	cg26671652	ZNF418	chr19:58446312	N_Shore	0.32	0.11	4.82E−11	3.20	2.18–4.39	0.58	0.93	0.79
	cg27062795	ZNF542	chr19:56879613	Island	0.43	0.12	1.23E−12	2.55	1.77–3.50	0.72	0.82	0.83

The sensitivity and specificity, as well as AUC, were both with a logistic regression prediction model without adjustment for gender, age, and smoking status and alcohol status

*Sens* sensitivity, *Spec* specificity, *AUC* area under the curve

^a^NA indicated that the CpG site is located outside of the coding region of the gene

^b^McaM represents the mean methylation percentage of the cases, and the McoM represents the mean methylation percentage of the controls

^c^ *P* value is calculated through the Wilcoxon rank-sum test followed by FDR (false discovery rate) adjustment for multiple correction

^d^OR and 95% CI were determined by logistic regression

**Table 3:** The mean methylation status of the five genomic regions in the validation datasets

Genomic region^a^	No. CpG sites^b^	CpG site included	Gene	McaM^c^	McoM^c^	*P* value^d^	log_10_(OR)^e^	95% CI^e^	Sens	Spec	AUC
chr8:99952469-99952722	19	cg15830431	STK3	0.35	0.16	4.20E−09	2.82	1.83–4.03	0.64	0.82	0.76
chr19:40314817-40314928	6	cg19396867	NA	0.36	0.12	9.60E−11	2.90	1.97–4.03	0.61	0.90	0.79
chr19:40314939-40315133	17	cg20655070	NA	0.31	0.12	1.80E−09	3.61	2.42–5.06	0.60	0.90	0.77
chr19:58446187-58446437	19	cg26671652	ZNF418	0.50	0.26	1.10E−13	3.46	2.52–4.54	0.74	0.86	0.84
chr19:56879517-56879735	25	cg27062795	ZNF542	0.41	0.14	5.20E−13	2.81	1.94–3.86	0.71	0.84	0.83

The sensitivity, specificity as well as the AUC were both with a logistic regression prediction model without adjustment for gender, age and smoking status and alcohol status

*Sens* sensitivity, *Spec* specificity, *AUC* area under the curve

^a^Genomic region represents the genomic coverage of the reads with targeted bisulfite sequencing, and the genomic coordinates shown here is based on the hg19 version of the genome

^b^No. CpG sites represents the number of the CpG sites in each region

^c^McaM represents the mean methylation percentage of the cases in each region, which consists of several CpG sites, while the McoM represents the mean methylation percentage of the controls in each region

^d^ *P* value is calculated through the Wilcoxon rank-sum test following with FDR (false discovery rate) adjustment for multiple correction

^e^OR and 95% CI were conducted through logistic regression

### The diagnostic ability of the five genomic regions based on machine learning methods

In order to get a better estimation of the diagnostic ability of the selected biomarkers, several machine learning methods, including logistic regression, random forest (RF), supporting vector machine (SVM), neural network (NN), Naïve Bayes (NB), linear discriminant analysis (LDA), mixture discriminant analysis (MDA), and flexible discriminant analysis (FDA), were utilized to build the diagnostic models for ESCC classification. The mean methylation percentages of the CpG sites in each genomic region were utilized for analysis. The five-fold cross-validation method was also conducted to give a robust estimation of the performance of the models. As shown in Table 4, in the training stage, the sensitivity of all the models ranged from 0.63 to 0.76 and the specificity ranged from 0.77 to 0.89. The logistic regression model and the SVM model both performed well with regard to accuracy. In the testing stage, the sensitivity of the models ranged from 0.63 to 0.73 and the specificity ranged from 0.78 to 0.88. The SVM model again achieved the highest accuracy, indicating the robustness and effectiveness of the model. In addition, we found that the diagnostic performance was similar between the training and testing stage in all the models, suggesting the reliability of our results.

**Table 4:** Diagnosis accuracy, sensitivity, and specificity of different classification models with five-fold cross-validation

Methods	Train			Test		
	Sensitivity	Specificity	Accuracy	Sensitivity	Specificity	Accuracy
Logistic regression	0.75	0.89	0.82	0.73	0.86	0.79
Random forest	0.73	0.77	0.75	0.73	0.78	0.75
Supporting vector machine	0.74	0.89	0.82	0.73	0.87	0.80
Naïve Bayes	0.63	0.89	0.76	0.63	0.88	0.75
Neural network	0.76	0.87	0.81	0.72	0.81	0.76
Linear discriminant analysis	0.73	0.88	0.80	0.71	0.87	0.79
Mixture discriminant analysis	0.74	0.89	0.81	0.71	0.84	0.77
Flexible discriminant analysis	0.73	0.88	0.80	0.71	0.87	0.79

The mean methylation percentage of each genomic region was considered as the independent variable for constructing the models, which means that all of the models were based on these five independent variables without adjustment for gender, age, smoking status, and alcohol status. Sensitivity, specificity, and classification accuracy were the mean value in five-fold cross-validations with 1000 replications

### Evaluation of diagnostic models in the ESCC subgroup analysis

Previous studies have found several risk factors for the incidence of ESCC, including age, gender, smoking status, and alcohol status [29–32]. As a result, we conducted subgroup analyses according to these risk factors. The mean methylation percentage of each targeted region was utilized for subgroup analysis. In the young/old subgroups, the median age of each patient was taken as the criteria for dividing the samples. We found that there was no significant difference between the sensitivity, specificity, and the AUC between the two subgroups (Additional file 2: Table S2). The overall AUC using all the variables in the two subgroups was 0.86 for both (Additional file 4: Figure S3A-B). In the male/female subgroups, we found that the diagnostic model performed better for the female subgroup than the male subgroup (Additional file 2: Table S3), and the overall AUC of the female subgroup was much higher than that of the male subgroup (AUC 0.89 vs. 0.84, Additional file 4: Figure S3C-D). In addition, in the smoker/non-smoker subgroup analysis, there was no significant difference between the diagnostic performances (Additional file 2: Table S4 and Additional file 4: Figure S3E-F). However, a significant difference was identified in the analysis of the alcohol/non-alcohol subgroups. Compared with the alcohol use subgroup, the AUCs in four of the five genomic regions were elevated in the non-alcohol subgroup, especially the two genomic regions covering *ZNF418* and *ZNF542* (Additional file 2: Table S5). The overall AUC obtained with all the genomic regions of the non-alcohol subgroup was substantially higher than that of the alcohol subgroup (0.89 vs. 0.79, respectively; Additional file 4: Figure S3G-H). In addition, we found that our female samples were all included in the non-alcohol subgroup. In order to eliminate the confounding factor of gender in the alcohol/non-alcohol subgroup analysis, we then selected the male samples only for subgroup analysis. Concordantly, we found that the diagnostic ability was still substantially better in the non-alcohol subgroup than in the alcohol subgroup, indicating that the observed difference was not introduced by the gender bias (0.90 vs. 0.79, respectively; Additional file 2: Table S6). The vast difference in the diagnostic performance in the alcohol/non-alcohol subgroup indicates that alcohol use may contribute to the epigenetic changes in ESCC as well as to the pathogenesis of ESCC [30].

## Discussion

DNA methylation plays a key role in the gene expression regulation and therefore has great potential as a non-invasive biomarker for cancer diagnosis and prognosis. ESCC patients who receive an early diagnosis will have longer survival times and lower mortality. Previous studies have found several candidate methylation biomarkers for ESCC detection and prognosis as well as treatment response. In our study, we integrated the methylation dataset from TCGA project and the GEO dataset for biomarker discovery and removed the candidate biomarkers with hyper-methylation status in PBMC and PBL cells of healthy controls to ensure its validity for future non-invasive diagnosis. Finally, a novel DNA methylation biomarker panel consisting of five CpG sites was then identified. Moreover, we validated these five CpG sites in 94 pairs of ESCC tumors and their adjacent normal tissues from Chinese patients with a targeted bisulfite sequencing method, enabling us to not only detect the methylation status of five CpG sites but their genomic regions as well. As a result, we then obtained the mean methylation percentage of each targeted region, which is a more robust estimation of the methylation status than the single CpG site itself. The methylation testing of these five genomic regions has a fairly high accuracy, sensitivity, and specificity in different models, suggesting that the methylation testing of these five genomic regions may be promising biomarkers for the detection of ESCC. In addition, the subgroup analyses identified that the diagnostic performance of the methylation testing is much better in the non-alcohol-consuming patients than in the ESCC patients who consume alcohol, suggesting the importance of taking the epidemiological data into considerations when performing ESCC diagnosis. Further studies may be required to explore the association between the methylation status of these five genomic regions and the use of alcohol.

Of the five genomic regions, two genomic regions covering cg19396867 and cg20655070 were not in the regulatory regions of specific genes. However, the H3k4me3, H3k4me1, and H3k27ac status of these two regions from the ENCODE project showed that these regions might be associated with the enhancers, indicating that the regions might also have important regulatory functions (data not shown). In contrast, cg15830431 (*STK3*, CpG Island), cg26671652 (*ZNF418*, CpG Shore), and cg27062795 (*ZNF542*, CpG Island) were either in the CpG islands or the CpG shores of a gene. The serine/threonine kinase 3 (*STK3*) gene encodes a serine/threonine protein kinase and functions as a growth suppressor, which is one of the key components of the Hippo signaling pathway involving apoptosis. A previous study has found that the deletion of *STK3* in mouse liver results in tissue overgrowth and tumor development, demonstrating its importance in suppressing carcinogenesis [33]. Also, hyper-methylation of *STK3* has been found in soft tissue sarcoma as well as head and neck squamous cell carcinoma, which is in accordance with the present study [34, 35]. However, in our expression analysis with the RNA-seq dataset from TCGA, we found that the expression of *STK3* was upregulated in the ESCC tumor tissues, which is inconsistent with our assumptions and needs further analysis (Additional file 5: Figure S4). *ZNF418* (zinc finger protein 418) is a member of the zinc finger-containing transcription factor family, which has been implicated as critical regulators for development and diseases. *ZNF418* has been shown to be a transcriptional repressor, which may act as a negative regulator in the MAPK signaling pathway, and we also found the downregulation of *ZNF418* in the ESCC tumor tissues in TCGA dataset, indicating the possible activation of MAPK pathway by decreased expression of *ZNF418* in the ESCC pathogenesis (Additional file 5: Figure S4) [36]. *ZNF542* (zinc finger protein 542) is a pseudogene, which also may be involved in transcriptional regulation. Studies have found hyper-methylation of *ZNF542* in oropharyngeal squamous cell carcinoma and sporadic colorectal cancer [37, 38]. Moreover, a pan-cancer study analysis based on the TCGA methylation datasets identified the hyper-methylation status of *ZNF542* in 12 cancer types [39]. Moreover, the expression profiles of *ZNF542* were in accordance with the methylation status, which was significantly downregulated in the ESCC tumor tissues (Additional file 5: Figure S4).

Several studies have recently conducted a search for the miRNAs and metabolomics, as well as DNA methylation-based biomarkers for ESCC diagnosis. Zhou X et al. have found a panel consisting of six microRNAs in serum which could serve as the biomarker for ESCC diagnosis [40]. Moreover, miR-1246, miR-18a, miR-25,s and miR-21 were all validated as the promising diagnostic biomarkers for ESCC previously [41–44]. Several mRNA-based biomarkers were also confirmed as candidate biomarkers for ESCC [45–47]. Jing X et al. found that the urine metabolomics were the promising diagnostic biomarkers for ESCC [48]. As for the DNA methylation-based biomarkers, Hiroaki N et al. have found that *HOXB2* and *SEPT9* were two candidate diagnostic biomarkers for the prediction of lymph node metastasis of ESCC [49]. In addition, the methylation status of *PAX1*, *ZNF582*, *HIN1*, *TFPI-2*, *DACH1*, and *SOX17* were all reported as the candidate diagnostic biomarkers for ESCC [50, 51].

Compared with the other kinds of biomarkers, DNA methylation alterations may occur in advance of the alterations of mRNA and protein levels in the carcinogenesis thus might have a better early diagnosis potential. Here, in our study, we integrated the public high-throughput microarray datasets and applied the targeted bisulfite sequencing method to explore the methylation status of our candidate CpG sites as well as their adjacent genomic regions. With the fast development of next generation sequencing (NGS), the targeted bisulfite sequencing method is becoming the recommended method for methylation detection because of high accuracy and high throughput and cost-effectiveness. Previous studies have revealed that the adjacent CpG sites on the same DNA molecules would share similar methylation patterns due to the locally coordinated activities of the DNA methyltransferases (DNMTs) or ten-eleven translocation (TET) proteins, which are methylation haplotypes, epi-alleles, or epi-haplotypes [52–54]. Because of the increased CpG sites in the region, the methylation haplotypes may be less susceptible to the complex and random environment stimulus and would be a more stable representative for methylation quantification [55].

Till now, the majority of the ESCC patients were diagnosed at later stage, and conventional endoscopy is expensive and depends on the availability of specialist clinical expertise and the diagnostic accuracy is relatively low [56]. As a result, better diagnostic methods for ESCC are urgently needed. Recently, a novel method which involves swallowing a sponge on a string has been proposed for ESCC diagnosis [57, 58]. The sponge is then gently pulled back out, taking a sample of cells from the person’s esophagus. In this case, our panel could be served as the diagnostic biomarkers. In addition, with the need for non-invasive diagnosis soaring up, our diagnostic panel could also be utilized for the liquid biopsy for ESCC in coordination with the other kinds of biomarkers.

The early diagnosis of esophageal squamous cell carcinoma is challenging due to its high heterogeneity. A single biomarker by itself may not be adequate for accurate diagnosis, which suggests that a panel consisting of multi-biomarkers is essential. Though our DNA methylation-based biomarkers have reached a fair accuracy in distinguishing the ESCC tumors from normal tissues, some of the ESCC tumor tissues still remained misclassified. Integration analysis of multi-omics datasets, ranging from genomics and epigenomics, as well as proteomics, may reveal more heterogeneity in ESCC and identify more biomarkers for accurate non-invasive diagnosis.

## Conclusion

Integration analysis of ESCC high-throughput DNA methylation datasets from TCGA project and GEO database identified five hyper-methylated CpG sites as candidate biomarkers for ESCC diagnosis, which were further validated in an independent analysis of 94 pairs of ESCC tumors and normal tissues using the targeted bisulfite sequencing method. Methylation profiles of the five genomic regions covering cg15830431 (*STK3*), cg19396867, cg20655070, cg26671652 (*ZNF418*), and cg27062795 (*ZNF542*) may be effective DNA methylation-based testing for ESCC diagnosis.

## Methods

### Biomarker discovery based on the public datasets

Public high-throughput DNA methylation microarray datasets were searched, and the comprehensive methylation dataset of esophageal cancer from the TCGA project was the first obtained. There were 84 ESCC and 3 normal tissues in this level 3 dataset. In addition, we also found that there are 78 EAC and 13 adjacent normal tissues in the TCGA dataset (level 3). To increase the sample size for a more robust biomarker discovery, the adjacent normal tissues of the EAC and ESCC were combined for analysis. Finally, 84 ESCC as well as 16 normal tissues were obtained from TCGA for discovery analysis. In addition, a GSE record named GSE52826 was found, with 4 ESCC and 8 normal tissue samples, which were utilized for preliminary validation.

To strengthen the robustness of the candidate biomarkers, we conducted the differential methylation region (DMR) analysis (Additional file 6: Figure S5). We first took the adjacent six CpG sites as a methylation block, and the range of the block should be shorter than 1000 bp due to the low methylation linkage equilibrium. We then applied the sliding window methods according to the genomic position of each CpG site and slides one CpG site each time. Therefore, some CpG sites were overlapped in the adjacent DMRs. Finally, we extracted 105,673 methylation regions which fulfilled our criteria. After that, we calculated the methylation status between the ESCC and the control tissues for each DMR. In summary, we obtained 411 DMRs, covering 1355 unique candidate CpG sites based on our standards (McaM > 0.40, McoM < 0.20, FDR < 0.01, fold change > 2). Subsequently, these 1355 candidate CpG sites were further filtered based on their methylation status and 713 candidate CpG sites were still retained (McaM > 0.25, McoM < 0.20, Diff > 0.15, fold change > 2, FDR < 0.01). Simultaneously, the methylation differences of the candidate CpG sites in the GEO dataset were also obtained for further validation (McaM > 0.15, McoM < 0.15, Diff > 0.10, fold change > 2, *P* value < 0.05). After that, only 275 candidate CpG sites were included. Due to the fact that it is inevitable to contain DNAs from some of the peripheral blood cells when performing liquid biopsy, to reduce the noises brought by the methylation status of the peripheral blood cells, it is of great importance that the methylation rate of the candidate biomarker should be very low in the adjacent normal tissues as well as in the peripheral blood so that it can be used for non-invasive cancer diagnosis in the future. As a result, we then filtered the candidate CpG sites with high methylation percentage in the peripheral blood mononuclear cells (PBMC, *N* = 111) and peripheral blood leucocytes (PBL, *N* = 527) of the healthy normal samples from the GEO database (the PBMC dataset came from the GSE53045 dataset, and the PBL dataset was the combination of GSE36054 and GSE42861). In addition, the CpG sites located far from the CpG islands were also filtered out. Moreover, we further removed the CpG sites with SNPs in their primers and the CpG sites whose corresponding genes have been studied in ESCC carcinogenesis. In total, 175 candidate CpG sites were finally selected. Based on the CG percent, PolyT, and the number of SNPs in the primers of our targeted regions, we obtained the overall score representing the difficulty levels for all the candidate regions. In total, we selected the top five candidate regions with the best chances to be amplified and conducted in the multiple PCR experiment and removed the other candidate regions for further validation. Finally, five of our candidate biomarkers were selected for further validation: cg15830431, cg19396867, cg20655070, cg26671652, and cg27062795.

### Patients, samples, and DNA

ESCC samples and their paired adjacent normal tissues for validation study were obtained from the First Affiliated Hospital of Soochow University and Fourth Military Medical University between the years of 2011 and 2015. The patients who did not undergo any neo-adjuvant therapy before the surgery were recruited only. All tumor tissues were evaluated by pathologists and fulfilled the criteria of tumor percent > 50%. All procedures performed in this study were in accordance with the ethical standards of the institutional research committee and with the 1964 Helsinki declaration and its later amendments. The studies were approved by the institutional review boards of Soochow University at Jiangsu Province and Fudan University, Shanghai, China. Written informed consent was obtained from each study subject. In addition, all of the subjects were re-examined and confirmed by professional pathologists for histopathological diagnosis. All tissues were immediately frozen at − 80 °C after surgical resection. Face-to-face interviews were conducted by professional investigators with a comprehensive questionnaire, including clinical information on tobacco smoking, alcohol consumption, and family history. The smokers were defined as ever using the tobacco products at least once a day for 6 months, and the alcohol drinkers were defined as ever using the alcohol products at least once a week for 6 months.

### Targeted bisulfite sequencing assay

DNA extraction and bisulfite conversion were performed as previously described [59, 60]. Based on the genomic coordinates of the five candidate CpG sites, we carefully designed the primers in order to detect them in a panel (Additional file 2: Table S7). The net-PCR was performed firstly to amplify the targeted DNA sequence. Then, the designed DNA fragments were sequenced by Illumina Hiseq 2000. BSseeker2 is one of the most commonly used tools for analyzing the bisulfite sequencing results and was applied in our study for mapping bisulfite-treated reads as well as for methylation calling [61]. After calling methylation, we obtained the bisulfite conversion rate for each sample, and the samples with bisulfite conversion rate < 98% were firstly filtered out. After the preliminary analysis, we then calculated the average coverage as well as the missing rate for each CpG site. The CpG sites with average coverage less than 20× and/or with missing rate > 0.20 were further filtered out. In addition, the samples with missing rate > 0.30 were filtered out finally.

### Statistical analysis and machine learning

In the discovery stage, we applied the Wilcoxon rank-sum test for testing the differential methylation status between cancer and normal tissues of each CpG site. Further, differential methylation status in tumor and normal tissues of the candidate CpG sites were tested with a logistic regression method. False discovery rate (FDR) correction was used for multiple test correction. In addition, the logistic regression (Package stats), support vector machine (SVM, Package e1071), random forest (Package randomForest), Naïve Bayes (Package e1071), neural network (Package nnet), linear discriminant analysis (LDA, Package mda), mixture discriminant analysis (MDA, Package mda), and the flexible discriminant analysis (FDA, Package mda) were used for classifying the ESCC and normal tissues. To obtain a robust evaluation of the prediction ability with these biomarkers and methods, five-fold cross-validation was also applied. In addition, sensitivity, specificity, and accuracy were obtained from the logistic regression model. All statistical analyses were all conducted using R 3.2.1 [62].

## Additional files

Additional file 1: Figure S1. PCA analysis of the ESCC and EAC adjacent normal tissues. (PDF 4 kb)

Additional file 2: Table S1. The methylation status of the five CpG sites in the GEO dataset and normal CD4^+^ and CD8^+^ T cells. Table S2 The methylation status of the five genomic regions in the young/old subgroups. Table S3 The methylation status of the five genomic regions in the male/female subgroups. Table S4 The methylation status of the five genomic regions in the smokers/non-smokers subgroups. Table S5 The methylation status of the five genomic regions in the alcohol/non-alcohol subgroups. Table S6 The methylation status of the five genomic regions in the alcohol/non-alcohol subgroups of male samples. Table S7 The designed primers of the five genomic regions for targeted bisulfite sequencing. (DOCX 51 kb)

Additional file 3: Figure S2. PCA analysis for the ESCC and adjacent normal tissues in the validation dataset. (PDF 6 kb)

Additional file 4: Figure S3. The ROC (Receiver Operating characteristics) curve for the subgroup analyzes. A-H represent the ROC curve for the young, old, male, female, smoked, non-smoked, alcohol, and non-alcohol subgroups, respectively. A-H each represent the overall ROC curve for the subgroup, which was calculated through a logistic regression model, incorporating the mean methylation percentage of the five genomic regions as the variables and without the adjustment for gender, age, and smoking status and alcohol status. (PDF 446 kb)

Additional file 5: Figure S4. The expression profiles for the three genes using RNA-seq data from TCGA. (TIFF 3006 kb)

Additional file 6: Figure S5. The detailed description of biomarker selection pipeline. (PDF 97 kb)

## Abbreviations

AUC: Area under the curve
EAC: Esophageal adenocarcinoma
ESCC: Esophageal squamous cell carcinoma
FDA: Flexible discriminant analysis
LDA: Linear discriminant analysis
MDA: Mixture discriminant analysis
NB: Naïve Bayes
NGS: Next generation sequencing
NN: Neural network
PBL: Peripheral blood cell
PBMC: Peripheral blood mononuclear cell
RF: Random forest
STK3: The serine/threonine kinase 3
SVM: Support vector machine
TCGA: The Cancer Genome Atlas
ZNF418: Zinc finger protein 418
ZNF542: Zinc finger protein 542
