# Supplementary figures and images for: Targeted bisulfite sequencing identified a panel of DNA methylation-based biomarkers for esophageal squamous cell carcinoma (ESCC)
Source: Clin Epigenetics. 2017 Dec 15;9:129. doi: 10.1186/s13148-017-0430-7 (PMC5732523; doi:10.1186/s13148-017-0430-7)

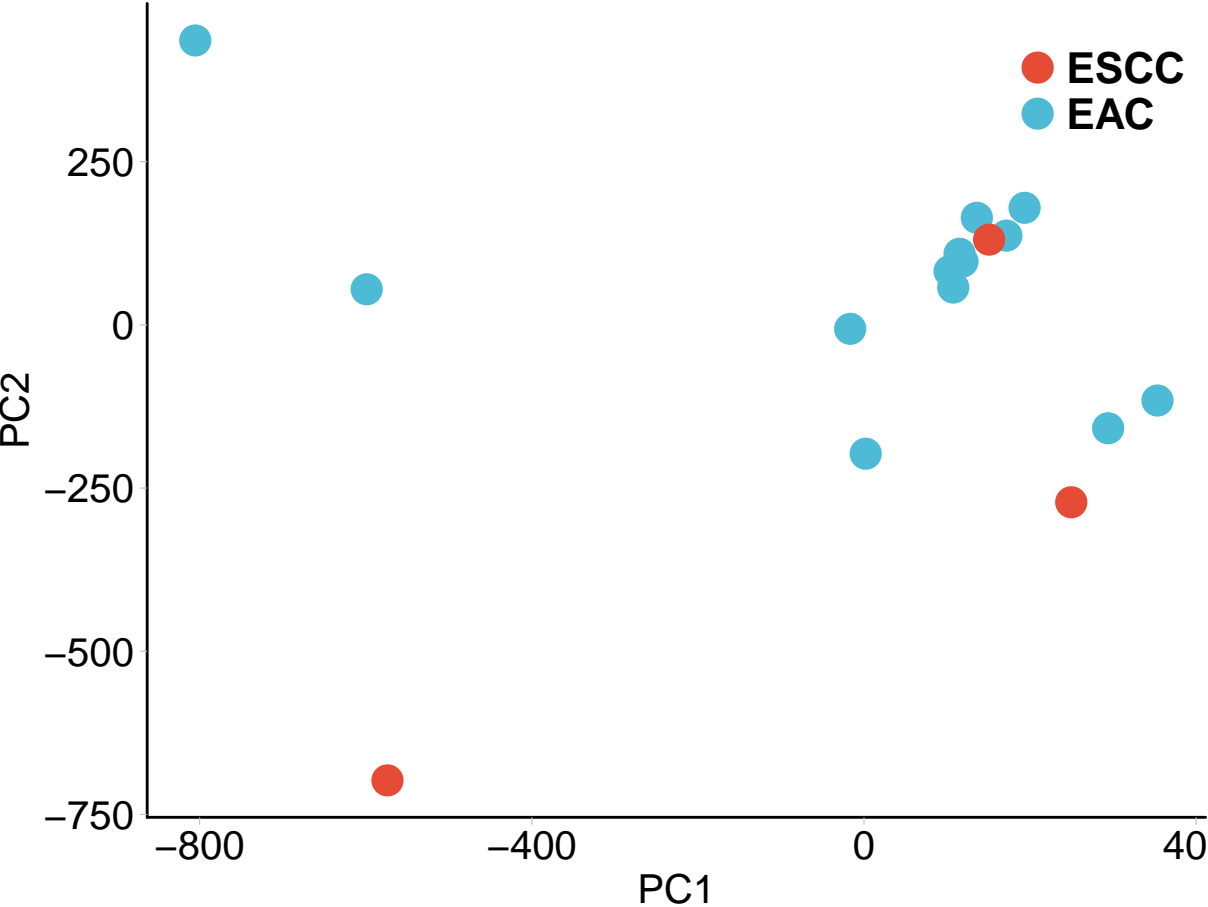

Supplement: Supplementary file 1 — PCA analysis of the ESCC and EAC adjacent normal tissues. (PDF 4 kb) [file 13148_2017_430_MOESM1_ESM.pdf]

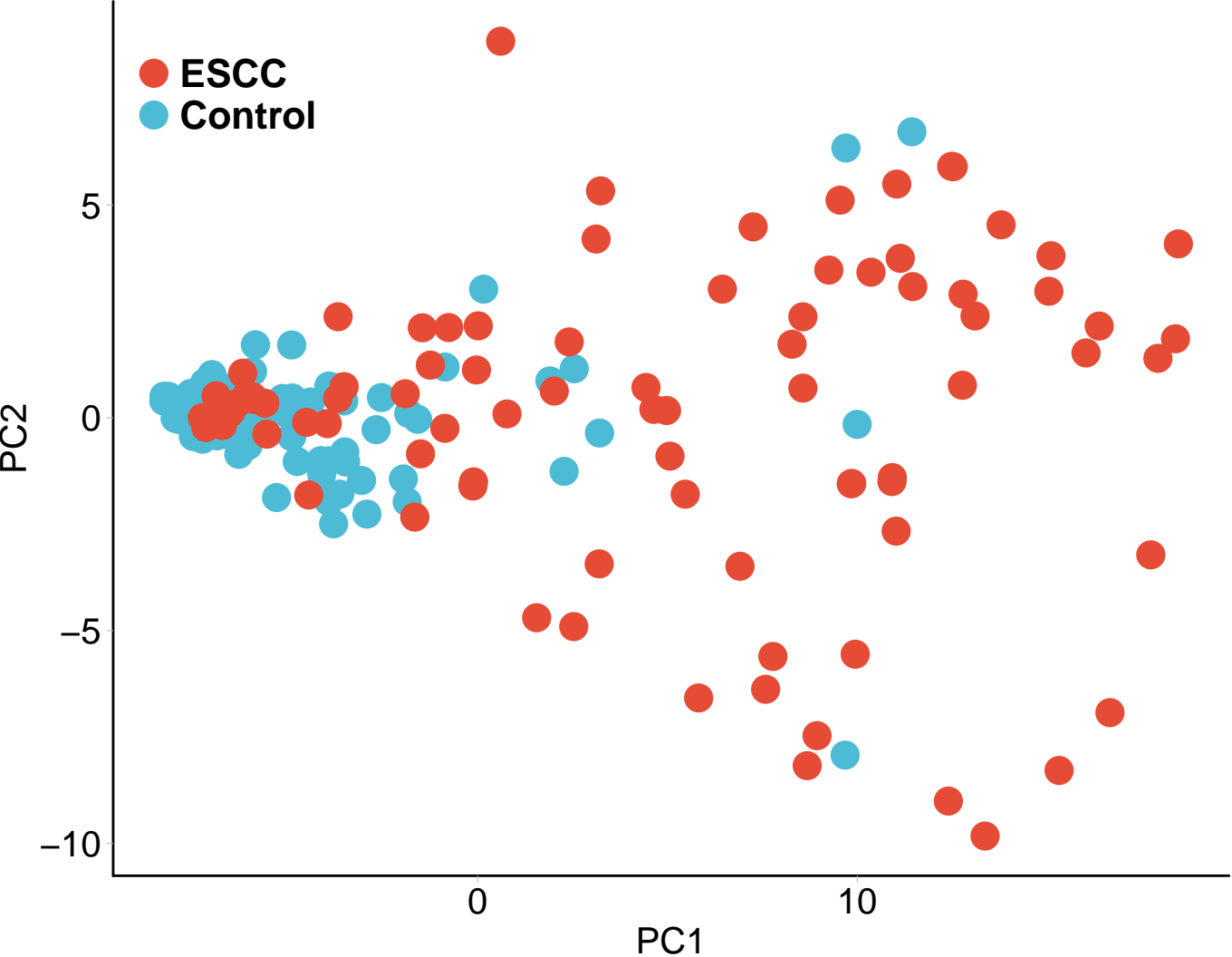

Supplement: Supplementary file 3 — PCA analysis for the ESCC and adjacent normal tissues in the validation dataset. (PDF 6 kb) [file 13148_2017_430_MOESM3_ESM.pdf]

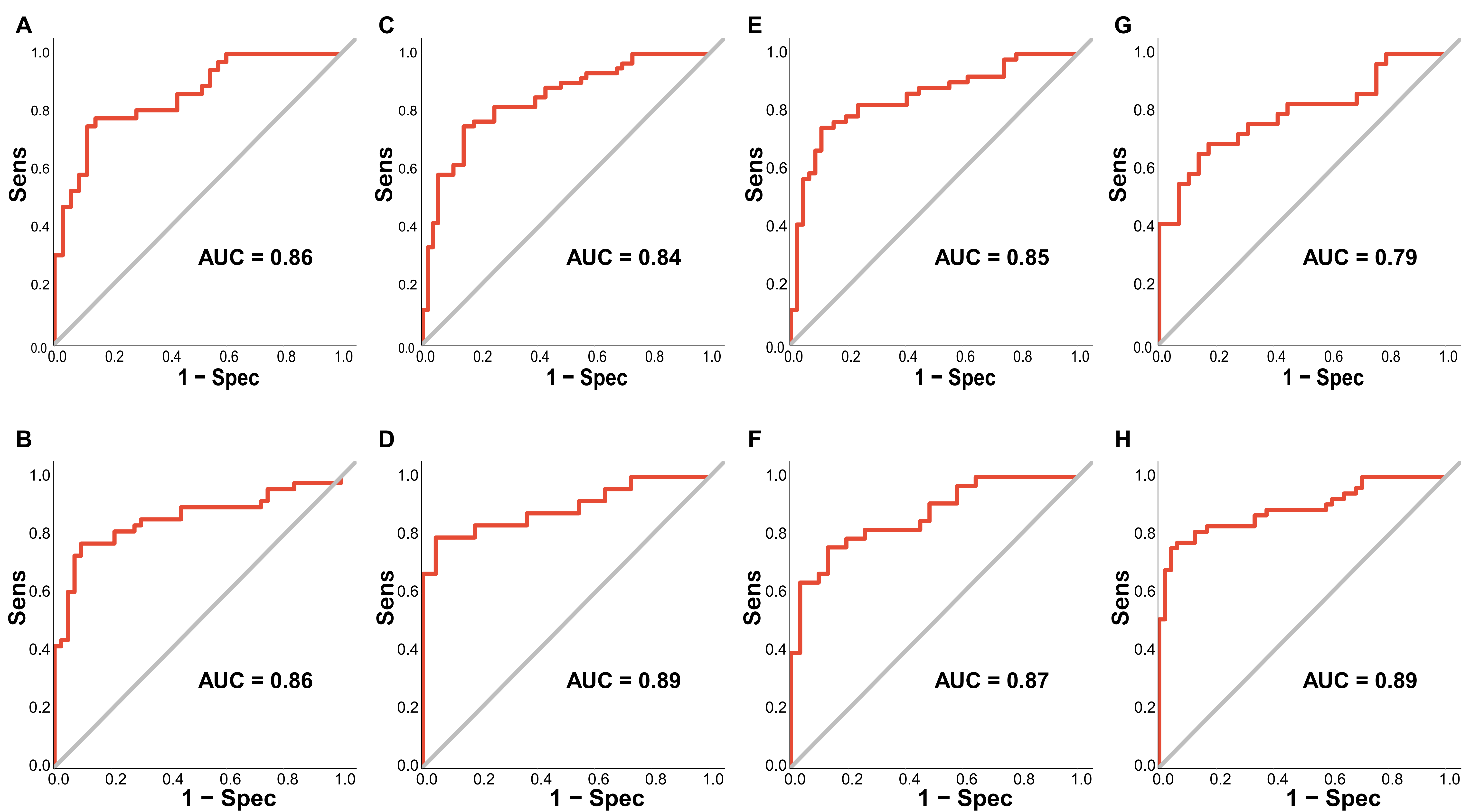

Supplement: Supplementary file 4 — The ROC (Receiver Operating characteristics) curve for the subgroup analyzes. A-H represent the ROC curve for the young, old, male, female, smoked, non-smoked, alcohol, and non-alcohol subgroups, respectively. A-H each represent the overall ROC curve for the subgroup, which was calculated through a logistic regression model, incorporating the mean methylation percentage of the five genomic regions as the variables and without the adjustment for gender, age, and smoking status and alcohol status. (PDF 446 kb) [file 13148_2017_430_MOESM4_ESM.pdf]

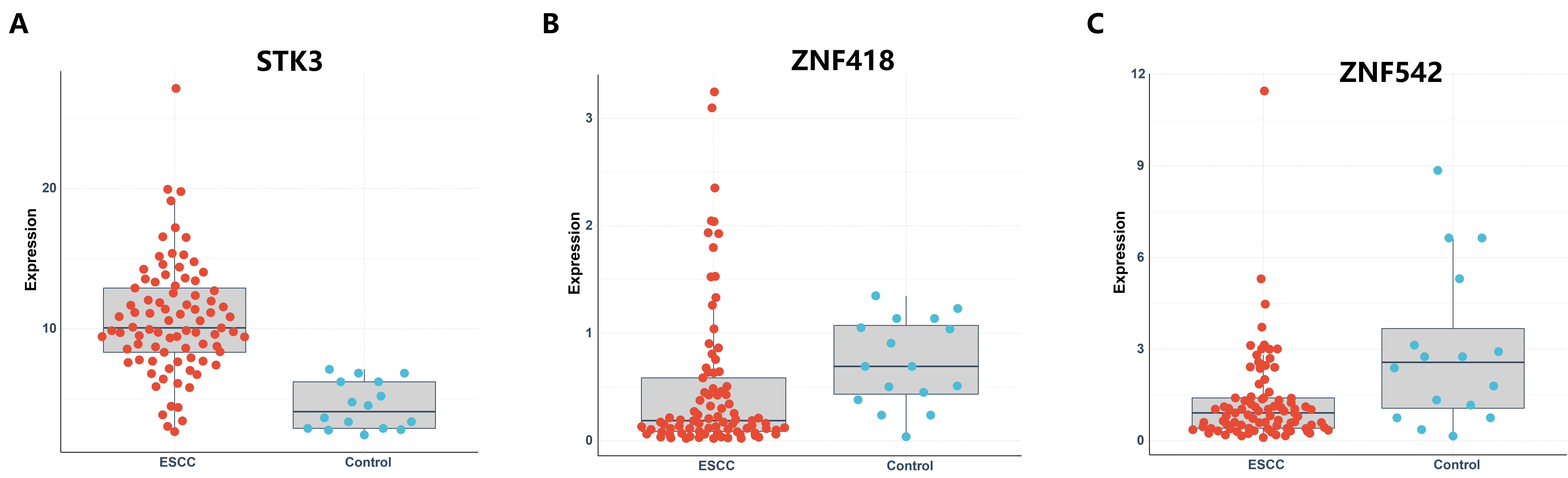

Supplement: Supplementary file 5 — The expression profiles for the three genes using RNA-seq data from TCGA. (TIFF 3006 kb) [file 13148_2017_430_MOESM5_ESM.tif]
