# Supplementary material for: Targeted bisulfite sequencing identified a panel of DNA methylation-based biomarkers for esophageal squamous cell carcinoma (ESCC)
Source: Clin Epigenetics. 2017 Dec 15;9:129. doi: 10.1186/s13148-017-0430-7 (PMC5732523; doi:10.1186/s13148-017-0430-7)
Supplement: Supplementary file 2 — The methylation status of the five CpG sites in the GEO dataset and normal CD4+ and CD8+ T cells. Table S2 The methylation status of the five genomic regions in the young/old subgroups. Table S3 The methylation status of the five genomic regions in the male/female subgroups. Table S4 The methylation status of the five genomic regions in the smokers/non-smokers subgroups. Table S5 The methylation status of the five genomic regions in the alcohol/non-alcohol subgroups. Table S6 The methylation status of the five genomic regions in the alcohol/non-alcohol subgroups of male samples. Table S7 The designed primers of the five genomic regions for targeted bisulfite sequencing. (DOCX 51 kb) [file 13148_2017_430_MOESM2_ESM.docx]

**Table S1 The methylation status of the 5 CpG sites in the GEO dataset and normal CD4^+^ and CD8^+^ T cells**

|  | **GSE52826 (N =12)** | | | | | | | | **PBMC (N=111)** | **PBL**  **(N=527)** |
| --- | --- | --- | --- | --- | --- | --- | --- | --- | --- | --- |
| **CpGsites** | **McaM^a^** | **McoM^b^** | **P value^c^** | **log_10_(OR)^d^** | **95% CI^d^** | **Sens^e^** | **Spec^e^** | **AUC^e^** | **MnoM^f^** | **MnoM^f^** |
| cg15830431 | 0.19 | 0.079 | 0.0283 | 14.7 | 2.49-41.2 | 1 | 0.75 | 0.91 | 0.07 | 0.08 |
| cg19396867 | 0.53 | 0.145 | 0.0202 | 14.2 | 1.71-76.5 | 1 | 0.75 | 0.94 | 0.05 | 0.09 |
| cg20655070 | 0.52 | 0.104 | 0.0067 | NA | NA | 1 | 1 | 1 | 0.14 | 0.12 |
| cg26671652 | 0.39 | 0.12 | 0.0067 | 237 | NA | 1 | 1 | 1 | 0.14 | 0.12 |
| cg27062795 | 0.46 | 0.078 | 0.0067 | 167 | NA | 1 | 1 | 1 | 0.10 | 0.08 |

McaM^a^ represent the mean methylation percentage of the ESCC samples in the GSE52826 dataset, and the McoM^b^ represent the mean methylation percentage of the control samples. The Pvalue^c^ is calculated through the Wilcoxon rank-sum test. log_10_(OR)^d^ and 95% CI^d^ were both obtained using logistic regression. Sens^e^ is short for sensitivity, and Spec^e^ is short for specificity, AUC^e^ is short for the area under curve, all of which were derived with the logistic regression prediction model without adjustment for gender, age and smoking status and alcohol status. MnoM^f^ represent the mean methylation percentage of the normal samples. The methylation data of the PBMC and PBL of the healthy normal samples were obtained from our unpublished dataset.

**Table S2 The methylation status of the 5 genomic regions in the Young/Old subgroups**

| **Subgroup** | **Gene^b^** | **Genomic** | **No.** | **McaM^e^** | **McoM^e^** | **Pvalue^f^** | **log(OR)^g^** | **95% CI^g^** | **Sens^h^** | **Spec^h^** | **AUC^h^** |
| --- | --- | --- | --- | --- | --- | --- | --- | --- | --- | --- | --- |
|  |  | **Region^c^** | **CpGsites^d^** |  |  |  |  |  |  |  |  |
| Young^a^  (N =45) | STK3 | chr8:99952469-99952722 | 19 | 0.38 | 0.16 | 5.80E-07 | 3.71 | 2.07-5.87 | 0.74 | 0.82 | **0.84** |
|  | cg19396867 | chr19:40314817-40314928 | 6 | 0.36 | 0.11 | 3.30E-06 | 3.53 | 1.99-5.63 | 0.72 | 0.85 | 0.81 |
|  | cg20655070 | chr19:40314939-40315133 | 17 | 0.31 | 0.11 | 1.10E-05 | 4.88 | 2.67-8.00 | 0.64 | 0.9 | 0.79 |
|  | ZNF418 | chr19:58446187-58446437 | 19 | 0.49 | 0.24 | 5.80E-07 | 3.44 | 2.07-5.16 | 0.71 | 0.9 | **0.84** |
|  | ZNF542 | chr19:56879517-56879735 | 25 | 0.4 | 0.13 | 3.30E-06 | 2.89 | 1.61-4.56 | 0.68 | 0.86 | 0.82 |
|  | Combined | - | - | - | - | - | - | - | **0.75** | **0.89** | **0.86** |
| Old^a^  (N = 49) | STK3 | chr8:99952469-99952722 | 19 | 0.32 | 0.16 | 2.00E-03 | 2.25 | 1.08-3.79 | 0.46 | 0.91 | 0.68 |
|  | cg19396867 | chr19:40314817-40314928 | 6 | 0.35 | 0.13 | 1.60E-05 | 2.51 | 1.40-3.91 | 0.56 | 0.91 | 0.76 |
|  | cg20655070 | chr19:40314939-40315133 | 17 | 0.3 | 0.13 | 3.80E-05 | 2.97 | 1.62-4.67 | 0.56 | 0.89 | 0.75 |
|  | ZNF418 | chr19:58446187-58446437 | 19 | 0.51 | 0.27 | 7.40E-08 | 3.51 | 2.27-5.01 | 0.77 | 0.83 | **0.84** |
|  | ZNF542 | chr19:56879517-56879735 | 25 | 0.42 | 0.14 | 8.40E-08 | 2.75 | 1.64-4.20 | 0.73 | 0.84 | **0.84** |
|  | Combined | - | - | - | - | - | - | - | **0.77** | **0.91** | **0.86** |

^a^The median ages of the patients were utilized as the criteria for dividing samples into the young and old groups. ^b^ cg19396867 and cg20655070 were in the non-coding region of the genome. ^c^Genomic region represents the genomic coverage of the reads with targeted bisulfite sequencing, and the genomic coordinates shown here were based on the hg19 version of the genome. ^d^No.CpGsites represents the number of the CpGsites in each region. ^e^McaM represents the mean methylation percentage of the cases in each region, which consists of several CpGsites, while McoM represents the mean methylation percentage of the controls in each region. ^f^P value is calculated through the Wilcoxon rank-sum test following with FDR (false discovery rate) adjustment for multiple correction. ^g^OR and 95% CI were calculated through logistic regression. ^h^Sens is short for sensitivty, while Spec is short for specificity, and the AUC is short for area under curve. The sensitivity, specificity as well as the AUC were both with a logistic regression prediction model without adjustment for gender, age and smoking status and alcohol status. The mean methylation percentage of each in each genomic region was considered as a variable.

**Table S3 The methylation status of the 5 genomic regions in the Male/Female subgroups**

| **Subgroup** | **Gene^a^** | **Genomic** | **No.** | **McaM^d^** | **McoM^d^** | **P value^e^** | **log(OR)^f^** | **95% CI^f^** | **Sens^g^** | **Spec^g^** | **AUC^g^** |
| --- | --- | --- | --- | --- | --- | --- | --- | --- | --- | --- | --- |
|  |  | **Region^b^** | **CpGsites^c^** |  |  |  |  |  |  |  |  |
| Male  (N =69) | STK3 | chr8:99952469-99952722 | 19 | 0.36 | 0.15 | 8.70E-09 | 3.39 | 2.09-5.06 | 0.66 | 0.85 | 0.81 |
|  | cg19396867 | chr19:40314817-40314928 | 6 | 0.34 | 0.12 | 3.10E-08 | 2.98 | 1.86-4.39 | 0.61 | 0.90 | 0.79 |
|  | cg20655070 | chr19:40314939-40315133 | 17 | 0.30 | 0.12 | 4.30E-07 | 3.69 | 2.27-5.49 | 0.60 | 0.90 | 0.76 |
|  | ZNF418 | chr19:58446187-58446437 | 19 | 0.50 | 0.27 | 6.40E-09 | 3.21 | 2.15-4.45 | 0.72 | 0.85 | **0.82** |
|  | ZNF542 | chr19:56879517-56879735 | 25 | 0.39 | 0.15 | 1.50E-08 | 2.49 | 1.54-3.64 | 0.70 | 0.82 | 0.81 |
|  | Combined | - | - | - | - | - | - | - | **0.75** | **0.86** | **0.84** |
| Female  (N = 25) | STK3 | chr8:99952469-99952722 | 19 | 0.33 | 0.17 | 9.94E-02 | 1.80 | 0.437-3.67 | 0.44 | 0.96 | 0.64 |
|  | cg19396867 | chr19:40314817-40314928 | 6 | 0.38 | 0.13 | 1.02E-03 | 2.75 | 1.23-4.90 | 0.60 | 0.96 | 0.78 |
|  | cg20655070 | chr19:40314939-40315133 | 17 | 0.33 | 0.13 | 1.34E-03 | 3.42 | 1.49-6.24 | 0.60 | 0.92 | 0.77 |
|  | ZNF418 | chr19:58446187-58446437 | 19 | 0.52 | 0.23 | 1.60E-05 | 4.25 | 2.34-6.89 | 0.80 | 0.88 | 0.88 |
|  | ZNF542 | chr19:56879517-56879735 | 25 | 0.47 | 0.11 | 1.60E-05 | 4.01 | 1.99-7.56 | 0.71 | 0.96 | **0.89** |
|  | Combined | - | - | - | - | - | - | - | **0.79** | **0.95** | **0.89** |

^a^ cg19396867 and cg20655070 were in the non-coding region of the genome. ^b^Genomic region represents the genomic coverage of the reads with targeted bisulfite sequencing, and the genomic coordinates shown here were based on the hg19 version of the genome. ^c^No.CpGsites represents the number of the CpGsites in each region. ^d^McaM represents the mean methylation percentage of the cases in each region, which consisted of several CpGsites, while the McoM represents the mean methylation percentage of the controls in each region. ^e^P value is calculated through the Wilcoxon rank-sum test following with FDR (false discovery rate) adjustment for multiple correction. ^f^OR and 95% CI were calculated through logistic regression. ^g^ Sens = sensitivity, while Spec = specificity, AUC = area under curve. The sensitivity, specificity as well as the AUC were both with a logistic regression prediction model without adjustment for gender, age and smoking status and alcohol status. The mean methylation percentage of each in each genomic region was considered as a variable.

**Table S4 The methylation status of the 5 genomic regions in the Smokers/Non-smokers subgroups**

| **Subgroup** | **Gene^b^** | **Genomic** | **No.** | **McaM^e^** | **McoM^e^** | **P value^f^** | **log_10_(OR)^g^** | **95% CI^g^** | **Sens^h^** | **Spec^h^** | **AUC^h^** |
| --- | --- | --- | --- | --- | --- | --- | --- | --- | --- | --- | --- |
|  |  | **Region^c^** | **CpGsites^d^** |  |  |  |  |  |  |  |  |
| Smoked^a^  (N =58) | STK3 | chr8:99952469-99952722 | 19 | 0.36 | 0.15 | 6.60E-08 | 3.81 | 2.23-5.90 | 0.68 | 0.87 | 0.81 |
|  | cg19396867 | chr19:40314817-40314928 | 6 | 0.34 | 0.11 | 4.00E-07 | 3.02 | 1.78-4.64 | 0.60 | 0.92 | 0.79 |
|  | cg20655070 | chr19:40314939-40315133 | 17 | 0.29 | 0.12 | 6.10E-06 | 3.86 | 2.23-5.99 | 0.58 | 0.92 | 0.76 |
|  | ZNF418 | chr19:58446187-58446437 | 19 | 0.51 | 0.27 | 3.20E-08 | 3.45 | 2.26-4.87 | 0.75 | 0.89 | **0.83** |
|  | ZNF542 | chr19:56879517-56879735 | 25 | 0.41 | 0.15 | 1.40E-07 | 2.65 | 1.61-3.93 | 0.71 | 0.81 | 0.81 |
|  | Combined | - | - | - | - | - | - | - | **0.75** | **0.89** | **0.85** |
| Non-smoked^a^  (N = 36) | STK3 | chr8:99952469-99952722 | 19 | 0.33 | 0.17 | 1.35E-02 | 1.92 | 0.72-3.46 | 0.56 | 0.79 | 0.67 |
|  | cg19396867 | chr19:40314817-40314928 | 6 | 0.38 | 0.13 | 4.53E-05 | 2.80 | 1.47-4.55 | 0.71 | 0.82 | 0.80 |
|  | cg20655070 | chr19:40314939-40315133 | 17 | 0.33 | 0.13 | 7.36E-05 | 3.36 | 1.73-5.52 | 0.71 | 0.79 | 0.78 |
|  | ZNF418 | chr19:58446187-58446437 | 19 | 0.50 | 0.24 | 1.60E-06 | 3.52 | 2.06-5.34 | 0.79 | 0.79 | 0.85 |
|  | ZNF542 | chr19:56879517-56879735 | 25 | 0.42 | 0.12 | 1.30E-06 | 3.18 | 1.69-5.43 | 0.67 | 0.94 | **0.87** |
|  | Combined | - | - | - | - | - | - | - | **0.76** | **0.87** | **0.87** |

^a^The smoker subgroup included the former smokers as well as current smokers. ^b^ cg19396867 and cg20655070 were in the non-coding region of the genome. ^c^Genomic region represents the genomic coverage of the reads with targeted bisulfite sequencing, and the genomic coordinates shown here were based on the hg19 version of the genome. ^d^No.CpGsites represents the number of the CpGsites in each region. ^e^McaM represents the mean methylation percentage of the cases in each region, which consisitng of several CpGsites, while the McoM represents the mean methylation percentage of the controls in each region. ^f^P value is calculated through the Wilcoxon rank-sum test following with FDR (false discovery rate) adjustment for multiple correction. ^g^OR and 95% CI were calculated through logistic regression. ^h^ Sens = sensitivity, while Spec = specificity, AUC = area under curve. The sensitivity, specificity as well as the AUC were both with a logistic regression prediction model without adjustment for gender, age and smoking status and alcohol status. The mean methylation percentage of each in each genomic region was considered as a variable.

**Table S5 The methylation status of the 5 genomic regions in the Alcohol/ Non-alcohol subgroups**

| **Subgroup** | **Gene^b^** | **Genomic** | **No.** | **McaM^e^** | **McoM^e^** | **P value^f^** | **log(OR)^g^** | **95% CI^g^** | **Sens^h^** | **Spec^h^** | **AUC^h^** |
| --- | --- | --- | --- | --- | --- | --- | --- | --- | --- | --- | --- |
|  |  | **Region^c^** | **CpGsites^d^** |  |  |  |  |  |  |  |  |
| Alcohol^a^  (N =34) | STK3 | chr8:99952469-99952722 | 19 | 0.32 | 0.17 | 4.20E-04 | 2.63 | 1.06-4.68 | 0.58 | 0.88 | 0.77 |
|  | cg19396867 | chr19:40314817-40314928 | 6 | 0.32 | 0.13 | 9.20E-04 | 2.63 | 1.14-4.64 | 0.58 | 0.88 | 0.75 |
|  | cg20655070 | chr19:40314939-40315133 | 17 | 0.28 | 0.13 | 3.80E-03 | 3.43 | 1.47-6.16 | 0.58 | 0.84 | 0.71 |
|  | ZNF418 | chr19:58446187-58446437 | 19 | 0.48 | 0.28 | 4.20E-04 | 2.88 | 1.46-4.58 | 0.67 | 0.91 | **0.78** |
|  | ZNF542 | chr19:56879517-56879735 | 25 | 0.37 | 0.15 | 4.20E-04 | 2.40 | 1.11-4.06 | 0.57 | 0.86 | **0.78** |
|  | Combined | - | - | - | - | - | - | - | **0.69** | **0.83** | **0.79** |
| Non-alcohol^a^  (N = 58) | STK3 | chr8:99952469-99952722 | 19 | 0.36 | 0.15 | 8.30E-06 | 2.92 | 1.67-4.57 | 0.57 | 0.91 | 0.75 |
|  | cg19396867 | chr19:40314817-40314928 | 6 | 0.36 | 0.11 | 5.50E-08 | 3.44 | 2.13-5.18 | 0.57 | 0.98 | 0.81 |
|  | cg20655070 | chr19:40314939-40315133 | 17 | 0.31 | 0.11 | 1.90E-07 | 4.22 | 2.57-6.41 | 0.61 | 0.94 | 0.79 |
|  | ZNF418 | chr19:58446187-58446437 | 19 | 0.51 | 0.23 | 1.50E-10 | 4.22 | 2.84-5.95 | 0.76 | 0.90 | **0.87** |
|  | ZNF542 | chr19:56879517-56879735 | 25 | 0.43 | 0.11 | 2.40E-10 | 3.70 | 2.32-5.64 | 0.74 | 0.90 | **0.87** |
|  | Combined | - | - | - | - | - | - | - | **0.75** | **0.96** | **0.89** |

^a^The Alcohol subgroup included the samples which had alcohol intake currently and formerly. ^b^ cg19396867 and cg20655070 were in the non-coding region of the genome. ^c^Genomic region represents the genomic coverage of the reads with targeted bisulfite sequencing, and the genomic coordinates shown here were based on the hg19 version of the genome. ^d^No.CpGsites represents the number of the CpGsites in each region. ^e^McaM represents the mean methylation percentage of the cases in each region, which consisitng of several CpGsites, while the McoM represents the mean methylation percentage of the controls in each region. ^f^P value is calculated through the Wilcoxon rank-sum test following with FDR (false discovery rate) adjustment for multiple correction. ^g^OR and 95% CI were calculated through logistic regression. ^h^ Sens = sensitivity, while Spec = specificity, AUC = area under curve. The sensitivity, specificity as well as the AUC were both with a logistic regression prediction model without adjustment for gender, age and smoking status and alcohol status. The mean methylation percentage of each in each genomic region was considered as a variable.

**Table S6 The methylation status of the 5 genomic regions in the Alcohol/ Non-alcohol subgroups of male samples**

| **Subgroup** | **Gene^b^** | **Genomic** | **No.** | **McaM^e^** | **McoM^e^** | **P value^f^** | **log(OR)^g^** | **95% CI^g^** | **Sens^h^** | **Spec^h^** | **AUC^h^** |
| --- | --- | --- | --- | --- | --- | --- | --- | --- | --- | --- | --- |
|  |  | **Region^c^** | **CpGsites^d^** |  |  |  |  |  |  |  |  |
| Alcohol^a^  (N =34) | STK3 | chr8:99952469-99952722 | 19 | 0.32 | 0.17 | 4.20E-04 | 2.63 | 1.06-4.68 | 0.58 | 0.88 | 0.77 |
|  | cg19396867 | chr19:40314817-40314928 | 6 | 0.32 | 0.13 | 9.20E-04 | 2.63 | 1.06-4.69 | 0.58 | 0.88 | 0.75 |
|  | cg20655070 | chr19:40314939-40315133 | 17 | 0.28 | 0.13 | 3.72E-03 | 3.43 | 1.06-4.70 | 0.58 | 0.84 | 0.71 |
|  | ZNF418 | chr19:58446187-58446437 | 19 | 0.48 | 0.28 | 4.20E-04 | 2.88 | 1.06-4.71 | 0.67 | 0.91 | **0.78** |
|  | ZNF542 | chr19:56879517-56879735 | 25 | 0.37 | 0.15 | 4.20E-04 | 2.40 | 1.06-4.72 | 0.57 | 0.86 | **0.78** |
|  | Combined | - | - | - | - | - | - | - | **0.69** | **0.83** | **0.79** |
| Non-alcohol^a^  (N = 33) | STK3 | chr8:99952469-99952722 | 19 | 0.39 | 0.13 | 8.00E-06 | 4.71 | 2.33-8.61 | 0.66 | 0.96 | 0.85 |
|  | cg19396867 | chr19:40314817-40314928 | 6 | 0.35 | 0.09 | 1.89E-05 | 4.55 | 2.31-8.22 | 0.62 | 0.96 | 0.83 |
|  | cg20655070 | chr19:40314939-40315133 | 17 | 0.29 | 0.10 | 5.19E-05 | 5.28 | 2.66-9.46 | 0.62 | 0.96 | 0.81 |
|  | ZNF418 | chr19:58446187-58446437 | 19 | 0.50 | 0.24 | 4.90E-06 | 4.19 | 2.35-6.72 | 0.72 | 0.93 | **0.87** |
|  | ZNF542 | chr19:56879517-56879735 | 25 | 0.39 | 0.12 | 4.40E-06 | 3.47 | 1.75-6.02 | 0.76 | 0.92 | 0.86 |
|  | Combined | - | - | - | - | - | - | - | **0.83** | **0.88** | **0.90** |

^a^The Alcohol subgroup included the male samples which had alcohol intake currently and formerly. ^b^cg19396867 and cg20655070 were in the non-coding region of the genome. ^c^Genomic region represents the genomic coverage of the reads with targeted bisulfite sequencing, and the genomic coordinates shown here were based on the hg19 version of the genome. ^d^No.CpGsites represents the number of the CpGsites in each region. ^e^McaM represents the mean methylation percentage of the cases in each region, which consisitng of several CpGsites, while the McoM represents the mean methylation percentage of the controls in each region. ^f^P value is calculated through the Wilcoxon rank-sum test following with FDR (false discovery rate) adjustment for multiple correction. ^g^OR and 95% CI were calculated through logistic regression. ^h^ Sens = sensitivity, while Spec = specificity, AUC = area under curve. The sensitivity, specificity as well as the AUC were both with a logistic regression prediction model without adjustment for gender, age and smoking status and alcohol status. The mean methylation percentage of each in each genomic region was considered as a variable.

**Table S7 The designed primers of the five genomic regions for targeted bisulfite sequencing**

| **Primer Name** | **Sequence** |
| --- | --- |
| cg15830431_F | GTTATTAGTTTGYGAGGTATTGAGTTATAGG |
| cg15830431_R | AAACCATCCTCACRCTCCAAAATC |
| cg19396867_F | GGATATTATTGGTYGTTGTGTTTGGTT |
| cg19396867_R | TCCATATTCACACAACAAACTATTTCTCTCTACCT |
| cg20655070_F | GTTTYGGGAGAGAGTGTYGGTTG |
| cg20655070_R | TCTCAACTCAAACAACTCCTAATCTTCC |
| ZNF418_F | GATTGTTTATAGGTTTGTAGTTYGGATT |
| ZNF418_R | CTCCTTTCTTCRAAACTCTCCATAA |
| ZNF542_F | TTTAGTTATTAGYGGAATTTTTTTTATTAGGTT |
| ZNF542_R | CTACCCACCCAAATCTCCCTAA |
