# Supplementary material for: Targeted bisulfite sequencing identified a panel of DNA methylation-based biomarkers for esophageal squamous cell carcinoma (ESCC)
Source: Clin Epigenetics. 2017 Dec 15;9:129. doi: 10.1186/s13148-017-0430-7 (PMC5732523; doi:10.1186/s13148-017-0430-7)
Supplement: Supplementary file 6 — The detailed description of biomarker selection pipeline. (PDF 97 kb) [file 13148_2017_430_MOESM6_ESM.pdf]

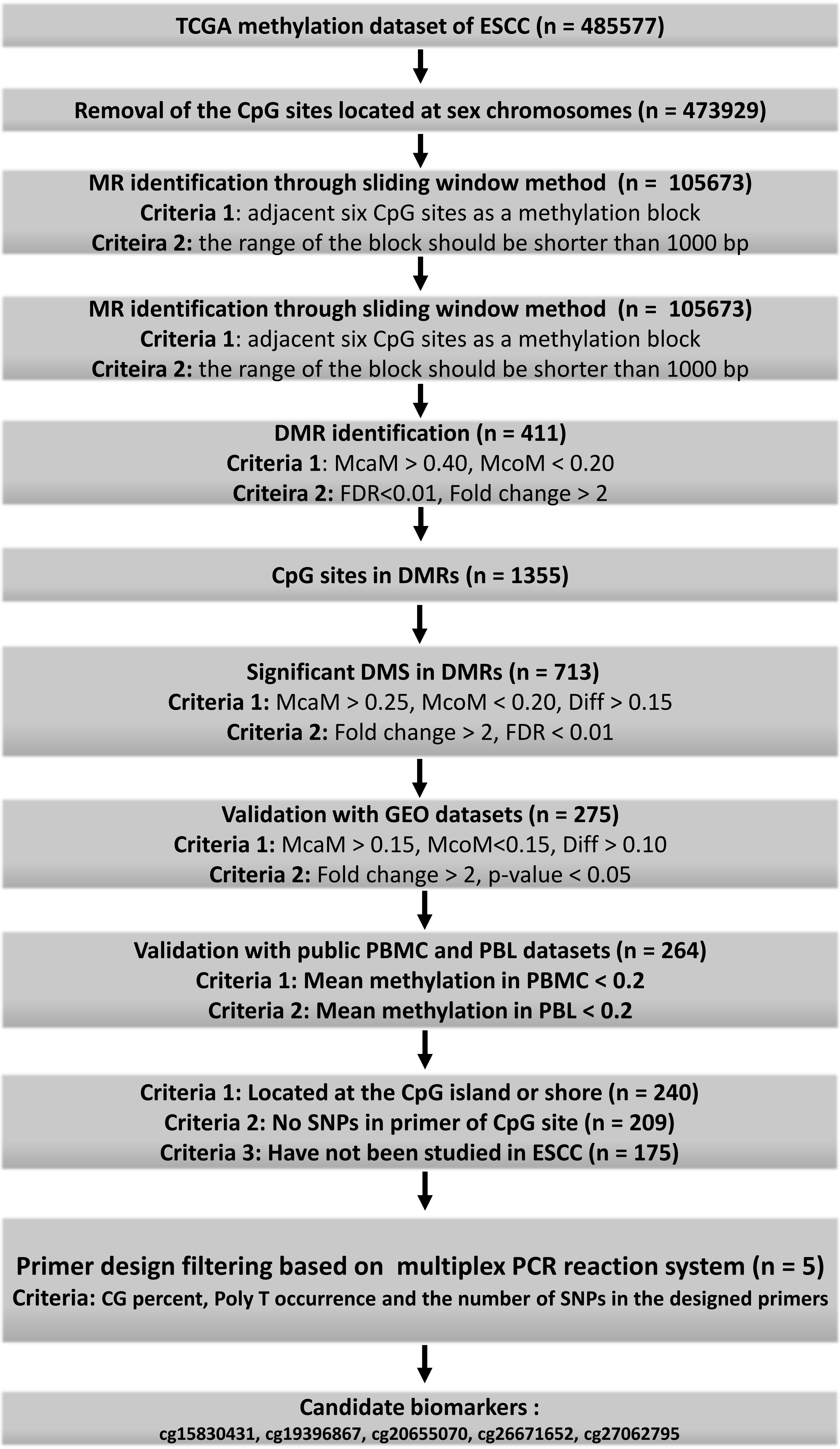
TCGA methylation dataset of ESCC (n = 485577)
Removal of the CpG sites located at sex chromosomes (n = 473929)
MR identification through sliding window method (n = 105673)
Criteria 1: adjacent six CpG sites as a methylation block
Criteira 2: the range of the block should be shorter than 1000 bp
MR identification through sliding window method (n = 105673)
Criteria 1: adjacent six CpG sites as a methylation block
Criteira 2: the range of the block should be shorter than 1000 bp
DMR identification (n = 411)
Criteria 1: McaM > 0.40, McoM < 0.20
Criteira 2: FDR<0.01, Fold change > 2
CpG sites in DMRs (n = 1355)
Significant DMS in DMRs (n = 713)
Criteria 1: McaM > 0.25, McoM < 0.20, Diff > 0.15
Criteria 2: Fold change > 2, FDR < 0.01
Validation with GEO datasets (n = 275)
Criteria 1: McaM > 0.15, McoM<0.15, Diff > 0.10
Criteria 2: Fold change > 2, p-value < 0.05
Validation with public PBMC and PBL datasets (n = 264)
Criteria 1: Mean methylation in PBMC < 0.2
Criteria 2: Mean methylation in PBL < 0.2
Criteria 1: Located at the CpG island or shore (n = 240)
Criteria 2: No SNPs in primer of CpG site (n = 209)
Criteria 3: Have not been studied in ESCC (n = 175)
Primer design filtering based on multiplex PCR reaction system (n = 5)
Criteria: CG percent, Poly T occurrence and the number of SNPs in the designed primers
Candidate biomarkers :
cg15830431, cg19396867, cg20655070, cg26671652, cg27062795
